# Hippocampus Maintains a Coherent Map Under Reward Feature–Landmark Cue Conflict

**DOI:** 10.3389/fncir.2022.878046

**Published:** 2022-04-26

**Authors:** Indrajith R. Nair, Guncha Bhasin, Dipanjan Roy

**Affiliations:** ^1^National Brain Research Centre (NBRC), Gurgaon, India; ^2^Centre for Brain Science and Application (CBSA), School of Artificial Intelligence and Data Science (AIDE), Indian Institute of Technology (IIT) Jodhpur, Karwar, India

**Keywords:** place cells, reward, attention, cue conflict, tetrode

## Abstract

Animals predominantly use salient visual cues (landmarks) for efficient navigation. When the relative position of the visual cues is altered, the hippocampal population exhibits heterogeneous responses and constructs context-specific spatial maps. Another critical factor that can strongly modulate spatial representation is the presence of reward. Reward features can drive behavior and are known to bias spatial attention. However, it is unclear whether reward features are used for spatial reference in the presence of distal cues and how the hippocampus population dynamics changes when the association between reward features and distal cues is altered. We systematically investigated these questions by recording place cells from the CA1 in different sets of experiments while the rats ran in an environment with the conflicting association between reward features and distal cues. We report that, when rewards features were only used as local cues, the hippocampal place fields exhibited coherent and dynamical orientation across sessions, suggesting the use of a single coherent spatial map. We found that place cells maintained their spatial offset in the cue conflict conditions, thus showing a robust spatial coupling featuring an attractor-like property in the CA1. These results indicate that reward features may control the place field orientation but may not cause sufficient input difference to create context-specific spatial maps in the CA1.

## Introduction

Animals use multimodal information to encode space, and the hippocampal system receives information from various sensory modalities (Ranck, 1973; Jacobs and Schenk, 2003). The hippocampal place cells use a repertoire of salient external sensory cues and internal network dynamics (attractor states) to organize the knowledge of any change in the environment (Tsodyks, 1999; Knierim and Zhang, 2012). Place field orientation is acutely controlled by distal cues (Muller and Kubie, 1987). In the absence of distal landmarks, local visual cues are used for context-specific spatial representation in the hippocampus (Draht et al., 2017). Many studies suggest that even macrosmatic animals mainly used distal landmarks for spatial and directional reference (Jeffery, 1998). Even though different sensory features like odor, sound, and taste are encoded in the hippocampus, they might be only used for spatial reference when the input from the visual modality is scarce (Zhang and Manahan-Vaughan, 2015; O’Keefe and Krupic, 2021). These studies indicate that the hippocampus predominantly uses visual cues as a reference frame to navigate the environment seamlessly (Cushman et al., 2013).

When animals encounter different environments, the hippocampus responds to them with a sparse, orthogonal representation, where different combinations of place cells are recruited to represent each environment (Alme et al., 2014). In addition, CA1 neurons exhibit heterogeneous responses in a dynamically changing environment where a fraction of place cells is controlled by distal cues while others are controlled by local cues (Lee et al., 2004). This mixed response, along with the remapping of place fields in different contexts, enables the hippocampus to establish environment-specific spatial maps. However, in most experiments, distal cues are visual, whereas local cues are multimodal (Shapiro et al., 1997; Knierim, 2002).

However, motivational stimuli like rewards drive the behavior and reorganize the spatial representation (Dupret et al., 2010) and may exert an intense evolutionary pressure to associate context and reward features for survival. Also, the learned association of visual stimuli with the reward is essential to shaping attention (Anderson, 2013; Chelazzi et al., 2013). Studies have shown that reward increases place field stability, modulates place cell discharge (Moser, 2004; Fenton et al., 2010), and biases hippocampal replay (Bendor and Wilson, 2012). Additionally, several studies have demonstrated that place fields cluster near the goal locations (Sosa and Giocomo, 2021). Also, the hippocampus contains a small subpopulation of cells that code only for reward (Gauthier and Tank, 2018). These reward cells resist remapping to any change in context, indicating a flexible representation of reward (Sosa and Frank, 2018). Thus, visual modality and reward features can be considered too strong and, sometimes, competitive factors in spatial navigation. Visual cues are predominantly used for spatial reference, whereas reward-related memories direct motivated behavior, attention, and decision-making (Sosa and Giocomo, 2021).

Nevertheless, it is unclear how hippocampal representation is driven by these two different cue systems when the association between the distal and reward features is altered. In the present study, we systematically investigated the dynamics of hippocampal representation in a dynamically changing reward feature-distal cue association by recording from the CA1 region of the dorsal hippocampus. We report that CA1 ensembles exhibited a highly coherent yet unstable spatial representation in the presence of reward features, thus exhibiting attractor network dynamics. In the absence of localized reward, the hippocampal neurons show orthogonal activity in a dynamically changing environment defined by distal cues and local textures, destabilizing the attractor network.

## Materials and Methods

### Subjects

Male Long-Evans rats (5–6 months old, *n* = 12) were housed separately on a reversed light-dark cycle (12:12 h). Food and water were provided *ad libitum*. All the experiments were conducted on the dark phase of the cycle. All procedures related to surgery, animal care, and euthanasia were approved by the Institutional Animal Ethics Committee (IAEC) of National Brain Research Center at Manesar, Haryana, constituted by the Committee for the Purpose of Control and Supervision of Experiments on Animals (CPCSEA), Government of India, and were conducted in accordance with NIH guidelines.

All the surgical procedures were performed under aseptic conditions. At the time of surgery, the rats were initially anesthetized with ketamine (60 mg/kg b.w.) and xylazine (8 mg/kg b.w.) and subsequently switched to isoflurane gaseous anesthesia. A custom-built microdrive containing 9–20 independently moving tetrodes (1–2 references in each microdrive) was surgically implanted over the right hemisphere to access dorsal CA1 of the hippocampus (coordinates 3.2–4.8 mm posterior to bregma, 1.2–2.8 mm lateral to the midline). To relieve pain, Meloxicam was administered intramuscularly (1 mg/kg b.w.) on the day of the surgery. During the post-surgical recovery period, Meloxicam was also provided through the oral route (1 mg/kg b.w.). During the post-surgical care of 7 days, food and water were provided *ad libitum*. The rats that were used for experiments using reward flavors were provided with different reward flavored pellets and checked for flavor preference. Following the recovery period, tetrodes were lowered into the brain to reach dorsal CA1 of the hippocampus. The rats were trained to run clockwise (CW) on a circular track to receive specific reward flavors at specific spokes on the track. During training and experimental sessions, the rats were maintained to no less than 85% of their body weight.

### Electrophysiological Recording

VG bonded platinum-iridium wire (17-μm) was used to make tetrodes (California Finw Wire, Grover Beach, CA, United States). The tips of individual wires were cleaned to remove debris by passing current (0.2 μA). Then, the tetrode tips were electroplated with the platinum black solution to reduce the impedance to 100–150 kΩ with 0.2 μA current. We used 96 channel data acquisition system to acquire electrophysiological recordings (Digital Lynx 10S, Neuralynx. Inc., Bozeman, MT, United States). The custom-made microdrive was attached to the EIB-27 board. The tetrodes and the references were connected to this EIB board. Headstage preamplifier (HS-27) was connected to the EIB-27 board. HS-27 was used to amplify the brain signals. HS-27 was connected *via* the commutator cables to the data acquisition system using specific tethers. The multi-unit activity was obtained by filtering the brain signals between 600 and 6 kHz. Spike waveforms were sampled for 1 ms at 32 kHz. The spikes waveforms were differentially recorded against a reference electrode in the corpus-callosum (a cell-free layer). Local field potentials (LFP) were differentially recorded against a ground screw on the skull above the frontal cortex. LFP signals were recorded as continuous signals with a bandwidth between 0.1 Hz and 1 kHz and sampled at 4 kHz. In this study, only the spike data are used. LFP signals were only used to visually observe ripples and complex spiking patterns, while the tetrode tips reached the hippocampus, and no analysis was performed on it. The rat position was tracked during experimental sessions using LEDs connected to the headstages, acquired at 25 Hz using a ceiling-mounted camera (CV-S3200, JAI Inc., San Jose, CA, United States.).

### General Experimental Design

After post-surgical recovery (∼7 days), the tetrodes were slowly advanced to reach the CA1 layer of the hippocampus over 12–21 days. The rats were trained for an average of 10 days (∼30 min/day) in a behavioral room to run clockwise (CW) on a centrally placed plain circular track/textured track (a 56-cm inner diameter, a 76-cm outer diameter) elevated 90 cm from the floor. Whenever the rat tried to turn back and run in a counter-clockwise (CCW) direction, the path was blocked using a cardboard sheet. A circular LED light mounted over the ceiling delivered the required illumination for the experiment room. A white noise generator placed centrally below the track apparatus was used to mask external noise and thus prevent any bias in behavior or/and neural activity due to any external noise. The experimental session as described below began once the tetrodes reached the CA1 region, and the animal was trained to complete 15–20 laps per session. Some tetrodes were adjusted each day to maximize the number of clusters recorded.

### Double Rotation Experiments With Textured Track as Local Cues (Tex)

Four rats were trained to run clockwise to fetch chocolate sprinkles placed at arbitrary positions on a textured circular track (inner diameter = 56 cm, outer diameter = 76 cm). The surface textures on the track served as local cues. There were four types of visually distinct textured surfaces (made of rubber sheet, carpet pad material, brown sandpaper, duct paper stripes), each enveloping the track’s quadrant. Six salient visual cues served as distal cues in which four cardboard cues were hung on a curtain, and two boxes of different sizes were positioned on the floor at the boundary of the curtain. Distal cues were entirely visual modality, whereas local cues were from visual and somatosensory modalities. This experimental paradigm precludes any representational bias due to reward anticipation as the reward was provided at random locations.

The Tex experimental paradigm followed the protocol shown in multiple studies (Knierim, 2002; Lee et al., 2004; Sharma et al., 2020). Each day of the experiments consisted of either five sessions (three standard sessions separated by two mismatch sessions) or three sessions (two standard sessions separated by one mismatch session). The cue configuration of the STD session is the same as that of the training sessions. In the mismatch sessions, cue conflict was created in which the distal cues were rotated clockwise (CW), and the textured circular track was rotated counterclockwise (CCW) by either 90° (MIS1) or 45° (MIS2).

### Double Rotation Experiments With Textured Track and Reward as Local Cues (Tex + Rwd)

The experimental paradigm is similar to the Tex experiments except that the reward is not placed randomly but at a particular location (at the junction of brown sandpaper and a black-textured cue). Each day of the experiments comprised of three standard sessions separated by two mismatch sessions. The cue configuration of the STD session is the same as that of the training sessions. In the mismatch sessions, cue conflict was created in which the distal cues were rotated CW, and the textured circular track was rotated CCW by either 90° (MIS1) or 45° (MIS2). The reward was placed at the exact location relative to surface textures in all the sessions. These experiments were conducted in the same four rats after completing double rotation experiments with the textured track as the only cues (Tex).

### Double Rotation Experiments With Different Reward Magnitude as Local Cues (RwdMag)

Each day of the experiment comprised of five sessions where three standard (STD) sessions were separated by a mismatch session (MIS1) and a flip session (FLIP). The configuration between the local cues (distinct reward magnitudes) and the distal cues in the STD sessions was preserved the same way as in the training sessions. The black circular track (a 56-cm inner diameter, a 76-cm outer diameter) had three visible spokes separated by 120° projecting outward from the track. The three spokes were separated by 120°. In STD sessions, the spoke that provided two pellets (high reward) was aligned (0°) with the stripe cue card, the spoke that provided no pellets (no reward) was aligned (0°) with the white cue card, and the spoke that provided one pellet (low reward) was aligned (0°) with the triangle cue card. In the MIS1 session, the track was rotated 120° CCW, and the distal cue was rotated 120° CW. In the FLIP session, the distal cues were not rotated. However, the spoke that provided high reward was provided with no reward, and the spoke with no reward was provided with high reward. Four rats were used in this experiment.

### Double Rotation Experiments With Different Reward Flavors as Local Cues (RwdFlav)

The black circular track is the same as that of RwdMag experiments. Three distinct flavors of reward pellets, namely, banana flavored, sucrose, and chocolate-flavored pellets, were provided at each spoke [Banana #F07257, Sucrose #F06233, Chocolate #07256, BioServ (Flemington, NJ, United States) Dustless Precision Pellets, 45 mg]. Each day of the experiment comprised of five sessions where three standard (STD) sessions were separated by two mismatch (MIS) sessions. The configuration between the local cues (distinct reward flavors) and the distal cues in the STD sessions was preserved the same way as the training sessions. In STD sessions, the banana flavor spoke was aligned (0°) with the stripe cue card, the sucrose flavor spoke was aligned (0°) with the white cue card, and the chocolate flavor spoke was aligned (0°) with the triangle cue card. In the first mismatch (MIS1) session, the track was rotated 60° CCW, and the distal cues were rotated 60° CW. This change in association led to a local-distal cue conflict due to a change in the configuration between the local cue (here, reward flavors) and the distal cues. In MIS1, the banana flavor spoke was aligned (0°) with a triangle cue card, the sucrose flavor spoke was aligned (0°) with a stripe cue card, and the chocolate flavor spoke was aligned (0°) with a white cue card.

All STD sessions have the same configurations. In the second mismatch session (MIS2), the track was rotated 120° CCW, and the distal cues were rotated 120° CW. This rearrangement led to a distinct configuration. Here, the banana flavor spoke was aligned (0°) with the white cue card, and the sucrose flavor spoke was aligned (0°) with the triangle cue card. The chocolate flavor spoke was aligned (0°) with the stripe cue card. The animals ran 15 laps in each session. There is no waiting time for the animal to receive the reward. Five animals were used in this experiment. Reward flavors act as local cues because the animals experience having rewards with distinct flavors at different spokes. Each flavor was exclusively available at a specific spoke only, i.e., one spoke received only one particular flavor of reward. The distal cues were visual, whereas local cues, here reward flavors, had distinct gustatory and olfactory features. Pilot experiments have shown that the rats differentiated reward flavors when they were restricted to 90–95% of their initial body weight. However, while the animal was near 85% b.w., no preference was observed, and we believe it might not have significantly impacted the results. Reward delivery systems were not intentionally used as they might act as a visual cue and may interfere with experiment results. Thus, rewards were provided manually. Distinct reward flavors were kept at the base of the track, hidden from the animals. To minimize the diffusion of odors, it was closed in three small boxes. Additionally, only 15–20 pellets were kept and were replenished after each session. These methods likely minimize odor diffusion during reward delivery. Moreover, fingers were cleaned regularly with water-based wet wipes to remove any mixing of odors while keeping rewards. After completing each session, the animals were provided with water so that the animals did not confuse between reward flavors due to saturation of taste buds and could identify reward flavors on the next session.

### Double Rotation Experiments With Different Reward Flavors and a Textured Track as Local Cues (RwdFlav + Tex)

In these experiments, the animals were trained in the STD configuration for just 2 days to familiarize themselves with the textured track as the animals already associated reward flavors with distal cues. This experiment was conducted to study whether the introduction of the textured track would stabilize standard sessions. The experimental paradigm is similar to RwdFlav experiments, except that the track is textured. The circular track had three different surface textures, each covering 1/3 of the track: a striped surface (black and white duct tapes), a black surface, and a rubber surface (blue colored). The center of the striped surface of the track was aligned with the banana flavor spoke, the center of the black surface was aligned with the sucrose flavor spoke, and the center of the rubber surface was aligned with the chocolate flavor spoke. The alignment of the distal cue and reward spoke was the same as in the RwdFlav experiment. Only the alignment between the textures and the spoke (reward flavor) was maintained when the track was rotated. The STD configuration is changed to create a local-distal cue conflict by rotating the textured track and reward flavors (CCW) and the distal cues (CW) by either 60° or 120° in MIS1 and MIS2 conditions, respectively. A total of 108 neurons from four animals were used in this experiment.

### General Experimental Procedure

At the start of the experiment, the rat was carried by the experimenter into the behavioral room inside a covered cardboard box to disrupt the animal’s sense of direction. The rat was then transferred on a pedestal placed at the center of the track. The tethers were connected to the EIB boards *via* the head stage. The rat was released at a random location on the track every session. After completing 15–20 laps, the rat was disconnected and transferred to the box for disorientation. During this period, the distal cues and the track were rotated to change the configuration. We repeated the same procedure for all the experiments. After completing all sessions on an experimental day, the circular track was wiped with 70% ethanol to remove any olfactory traces that could potentially bias the behavior and neural representation on the next day of recording. Rat droppings during experimental sessions, if any, were removed with tissue paper. In most of the animals, more than one experiment was conducted. Details of the experiments conducted on each rat are shown below.

**Table:** 

Rat #	Experiment order		
	1	2	3
R-33	Tex	Tex + Rwd	
R-47	Tex	Tex + Rwd	
R-58	Tex	Tex + Rwd	
R-A16	Tex	Tex + Rwd	
R-086	RwdFlav	RwdMag	
R-106	RwdFlav		
R-109	RwdMag		
R-113	RwdFlav	RwdMag	
R-119	RwdFlav	RwdMag	RwdFlav + Tex
R-123	RwdFlav	RwdFlav + Tex	
R-125	RwdFlav + Tex		
R-126	RwdFlav + Tex		

### Data Analysis and Statistical Tests

The data analysis and statistical tests were performed in Matlab (MathWorks R2019a), Circular Statistics (Oriana, Kovach Computing Services, United Kingdom), and Graph Pad Prism.

### Isolation of Single Units

All data analysis, including spike sorting, was performed offline. Single units from tetrode recordings were isolated using custom-written spike-sorting software (Winclust, Siegel et al., 2008; Sharma et al., 2020). Cells were manually isolated based on the spike parameters such as peak amplitude, energy, peak-to-valley amplitude recorded on four tetrode channels. Based on the cluster’s distance from the background, waveform shape, inter-spike interval (ISI), and potential overlap between neighboring clusters present, isolation quality was rated on a scale ranging from 1 to 5 (1–very good, 2–good, 3–fair, 4–marginal, 5–poor). All units rated “fair” and above were used for analysis. Marginal and poor clusters were excluded from the analysis. Clusters that had >1% spikes less than ISIs (<3 ms) were discarded. Neurons with a mean firing rate > 10 Hz were not used for analysis (putative interneurons). The cluster ratings were independent of the spatial property of the unit isolated. Only place cells were used in this study.

### Place Cell Identification

We used the following criteria to classify place cells:

1. Mean spike width > 200 μs (putative excitatory neurons, Okada et al., 2017).
2. A mean speed-filtered firing rate in at least one session between 0.05 and 10 Hz.
3. Have a statistically significant (*p* < 0.05, a re-randomization test using a shuffling procedure) spatial information score > 0.5 with a clear place field in at least one of the sessions on an experimental day.

A spatial information score indicates the amount of information carried about the rat’s spatial location by a cell (Skaggs et al., 1993). It was calculated according to the formula written and described below.


$$I=\sum_{x}\lambda \left(x\right)log_{2}\left(\frac{\lambda \left(x\right)}{\lambda }\right)p\left(x\right)$$

where I is the spatial information score, λ(*x*) is the mean firing rate in each pixel (position), λ is the mean firing rate of the cell, p(x) is the probability of the rat to be in the pixel x [occupancy time in pixel (x)/total occupancy time]. The spatial information score was computed on a smooth speed-filtered rate map.

### Rotational Correlation

In this study, analysis of hippocampal place cells was performed at different levels. Here, we used the terminology “ensemble” to indicate the simultaneously recorded neurons within an experimental day. The term “population” meant all the hippocampal neurons recorded across days (all neurons pooled). The population is further grouped based on the orientation of neuronal ensembles. To quantify the change in the firing field of place cells in the hippocampus, we conducted rotational correlation analysis on the ensemble recorded on each day of the recording. Two-dimensional occupancy and spike data were linearized to obtain a one-dimensional firing rate array of 72 bins each (5° each per bin). One-dimensional firing rate arrays were constructed by dividing the number of spikes fired when the rat was in a particular bin by the amount of time spent (in seconds) in that specific spatial bin on the circular track. To exclude behavioral and neural epochs when the rat was immobile (especially at the spokes), positional sampling in which the velocity of the rat was <1 cm/s was removed. We have quantified the rotation of the preferred firing field of individual cells between STD sessions or between STD and MIS sessions using Pearson’s product-moment correlation between linearized firing rate arrays of a cell between two corresponding sessions. We did this procedure 71 times by shifting the session being correlated by one bin increment (5°) to obtain shifted correlation (Neunuebel et al., 2013). The angle at which maximum correlation is obtained is regarded as the rotation of the place field between the sessions. Circular statistical tests were conducted on the ensemble and population data (grouped) to calculate the angle and length of the mean vector using Oriana Software (Kovach Computing Services, United Kingdom). The angle of the vector represents the mean angle of rotation of the place fields in the ensemble/population (grouped). The mean vector length (MVL) determines the compactness of the distribution of angles of the cells in the ensemble/population (grouped). It is inversely proportional to the variance of the distribution around the mean.

### Cluster Optimization

To identify any patterns in the mean angle of orientation across ensembles in different comparisons, we used the k-means elbow method to determine the optimum number of clusters based on the mean vector length and the mean angle of rotation of place fields from all the ensembles. The optimum number of clusters was found to be three across all the comparisons. We then used the k-means algorithm to group the ensembles based on their mean direction and mean vector length into three different clusters.

### Population Correlation Analysis

To measure the population activity of the hippocampus, 2D spatial correlation matrices were created from firing rate vectors (1D) at each position on the track (Neunuebel et al., 2013). Population correlation matrices for STD vs. STD and STD vs. MIS sessions were created for the grouped and pooled cells. Firing-rate arrays (1° bin) of pooled cells and grouped cells were separately combined to generate an N × 360 matrix where N is the number of cells and 360 is the total number of positional bins. A Pearson’s product-moment correlation analysis was performed between the standard session matrix and the subsequent standard or the mismatch session to create a 360 × 360 correlation coefficient matrix. The population correlation matrix comprises Pearson correlation values acquired from the bin-wise correlation of the 1D firing rate arrays. For STD vs. STD and STD vs. MIS comparisons, higher correlation angles would be expected at the angle equivalent to the rotation of the preferred direction of firing fields. Population responses were quantified by transforming 2D correlation matrices to 1D polar plots by calculating the average correlation of pixels in each of the diagonals in the correlation matrices. The angle that showed the maximum correlation is regarded as the amount of rotation of the preferred direction of the firing field of the hippocampal population.

### Spatial Cross-Correlation

One of the characteristic features of attractor dynamics in the spatial neural network is the maintenance of the spatial offset between place fields of co-recorded cells. We studied the attractor dynamics of the hippocampus in different experiments by comparing the spatial offset between co-recorded place cells across standard and mismatch sessions. We modified the analysis performed by Bassett et al. (2018) so that spatial cross-correlation (SXC) was performed for the entire session duration (Sharma et al., 2020), as described below. Two-dimensional spike and occupancy data were linearized to produce 1D firing rate arrays of bin size 5° (total, 72 bins). The firing rate arrays were divided by their peak value to create normalized firing rate arrays. To quantify the spatial offset of the SXC arrays, polar plots for each SXC cell pair (72 bins) were created, and the mean direction of the polar plot was determined using a MATLAB-based circular statistics toolbox (Berens, 2009). The mean direction value of a cell pair in a particular session represents its spatial offset in that session. The coupling between cell pairs in the population was quantified by correlating the spatial offset values (after correcting for −180°/180° transition values) between the subsequent sessions by calculating the correlation coefficient values and their statistical significance.

### Histological Analysis

Marker lesions were made on a selected few tetrodes by passing current (10 μA for 10 s). The rats were perfused transcardially on the following day using 4% formaldehyde solution. After perfusion, the brain was extracted and preserved in 30% sucrose-formalin solution till it sank. Later, brains were sectioned (Coronal plane, 40-μm sections), mounted, and left to dry. Sections were then stained through the Nissl stain procedure using 0.1% Cresyl Violet. Images of the sections were acquired using a Leica DFC265 digital camera connected to a Leica M165-C stereomicroscope. The tetrodes on the sections were identified using tetrode arrangements in the microdrive. Finally, depth reconstruction was conducted on each tetrode track to identify the brain region from which electrophysiological recordings were conducted each day based on the tetrode tip distance. We also considered the shrinkage factor (15%) of the brain tissue due to histological processing.

## Results

**FIGURE 1:**
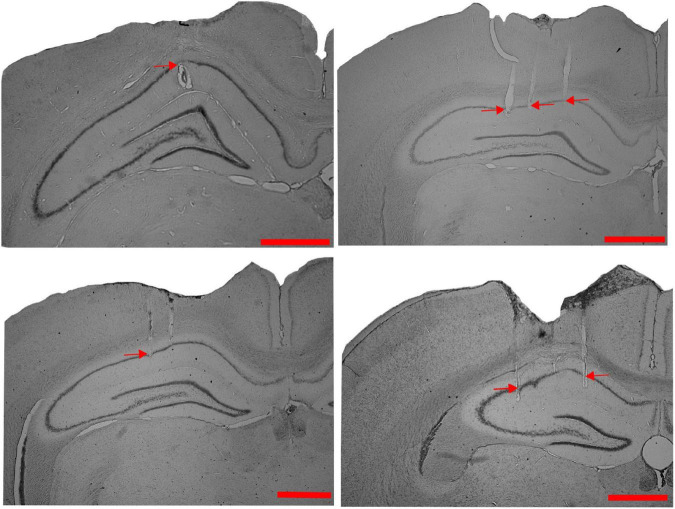
Tetrode localization in CA1. Representative examples of Nissl-stained coronal sections from four different rats, showing tetrode tracks (indicated by red arrows) in the CA1 region of the hippocampus (Scale bar = 1 mm).

We studied the dynamics of the spatial representation in the hippocampal CA1 population when the animals experienced a change in the association between reward features (with or without other local cues) and distal cues in the dynamically changing environment in different sets of experiments. Figure 1 shows the representative examples of tetrode localization in the Nissl stained coronal sections of hippocampal CA1 from four different rats.

### Double Rotation Experiments With the Textured Track as Local Cues (Tex)

**FIGURE 2:**
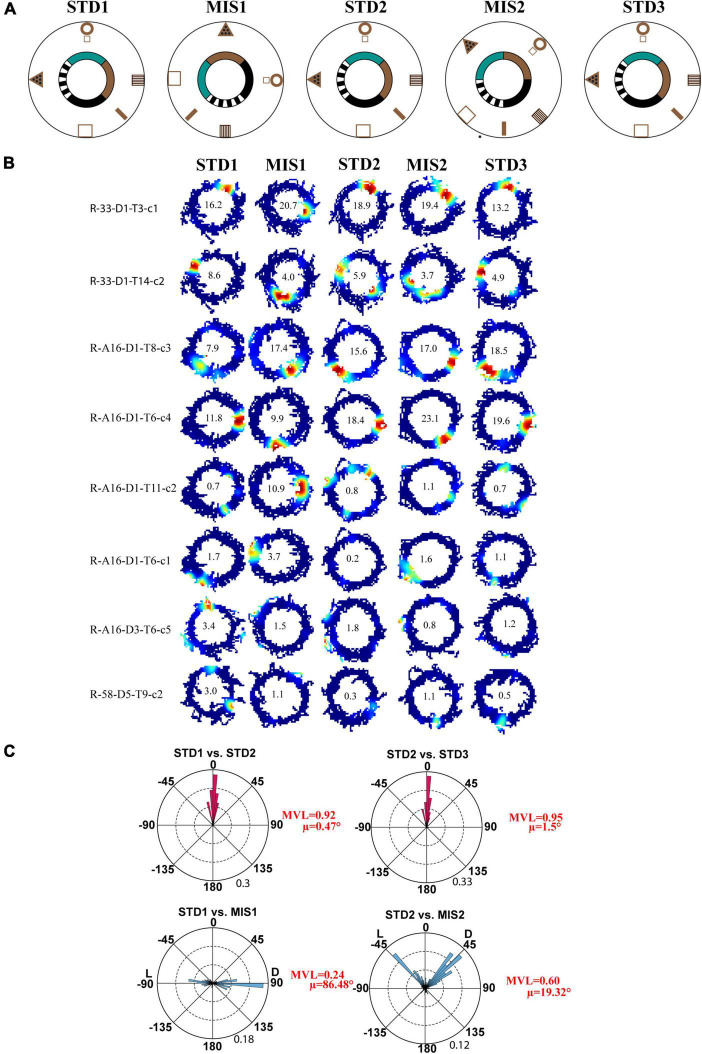
Schematic representation of the double rotation experiments with a textured track as a local cue (Tex). **(A)** The schematic representation of the Tex double-rotation paradigm. In this paradigm, the reward was provided at random locations on the textured track. **(B)** Representative place fields from five sessions showing heterogeneous representation. The number inside the firing rate maps indicates the peak firing rate [in Hertz (Hz)]. The rate maps are color coded, with red color denoted by >90% of the peak firing rate; no firing is indicated by blue, and successive gradients in the firing rates are shown with intervening colors in the spectrum. Clusters R-33-D1-T3-c1 and R-33-D1-T14-c2 are co-recorded neurons orienting to distal and local cues, respectively, demonstrating heterogeneous response from CA1. Clusters R-A16-D3-T6-c5 and R-58-D5-T9-c2 show the place field that did not fire in all sessions. Cluster R-A16-D1-T11-c2 shows remapping of place cells. **(C)** Circular histogram shows place field rotation across different standards and mismatch comparisons. L and D indicate the rotation of local and distal cues. The mean vector length (MVL) and mean direction (μ) of comparisons are shown alongside the plot. The numbers in brown indicate the maximum proportion of place fields (an outer circle).

First, we replicated cue-conflict experiments using the classical double rotation protocol (Knierim, 2002). In this experiment, surface textures on the track were considered the local cues, and reward was provided at random locations to motivate the animals to run CW on the circular track (Figure 2A). We recorded 232 CA1 place cells from four animals to observe the hierarchical representation of hippocampal neurons to cues. Figure 2B shows the representative examples of hippocampal place fields from co-recorded neurons simultaneously binding to distal and local cues, suggesting heterogeneous response. Figure 2C shows the circular histogram of STD vs. MIS and STD vs. STD comparisons. Place fields show stability in the STD sessions, exhibiting a significant mean vector length [MVL_(STD1 vs. STD2)_ = 0.92, MVL_(STD2 vs. STD3)_ = 0.95, Rayleigh’s test *p* < 0.01 in both comparisons]. During both mismatch sessions, place fields were oriented to either local or distal cues [MVL_(STD1 vs. MIS1)_ = 0.24, MVL_(STD2 vs. MIS2)_ = 0.60, Rayleigh’s test *p* < 0.01 in both comparisons]. These results replicate findings from other studies that CA1 exhibits heterogeneous representation (Knierim, 2002; Lee et al., 2004; Yoganarasimha et al., 2006).

### Double Rotation Experiments With Textured Track and Reward as Local Cues (Tex + Rwd)

**FIGURE 3:**
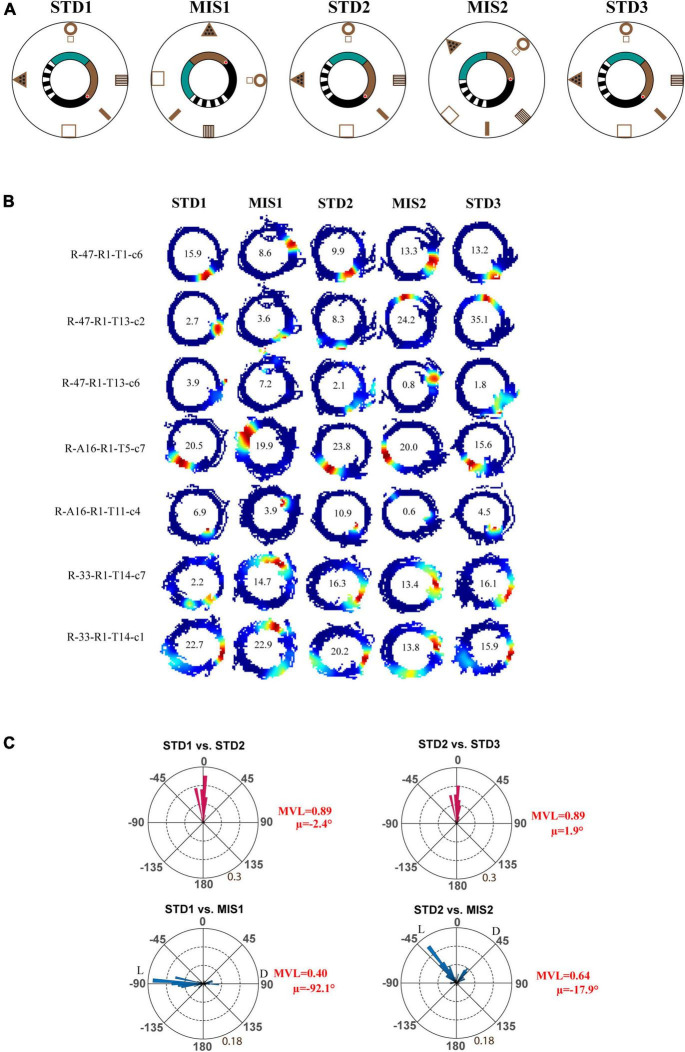
Schematic representation of the double rotation experiments with a textured track and reward as local cues (Tex + Rwd). **(A)** The schematic representation of a double-rotation paradigm in which reward was provided at a particular location on the textured track. A white dot with the red center indicates the reward location. **(B)** Representative place fields from different experimental days from five sessions. The numbers inside the firing rate maps indicate the peak firing rate in Hz. The rate maps are color coded as described in Figure 2. **(C)** Circular histogram shows place field rotation of hippocampal population across different standards and mismatch comparisons. The corresponding mean vector length (MVL) and mean direction (μ) of each comparison are shown alongside the plot. L and D indicate the rotation of local and distal cues.

Next, we checked whether localizing reward on a particular location alters the hierarchical representation of the space by the hippocampus. In the Tex experiment, the reward was provided at random locations to motivate the animals to run CW on the track. But, in this experimental paradigm, the reward (the chocolate sprinkle) was provided only at one specific location on the circular track. The experimental paradigm is shown in Figure 3A. Each day of the experimental session comprises three standard (STD) sessions separated by two mismatch (MIS) sessions. In the MIS session, the textured track was rotated CCW, and the distal cues were rotated CW by either 90° (MIS1) or 45° (MIS2), leading to a change in the association between local and distal cues. We recorded 117 CA1 place cells from four animals in this experiment. Figure 3B shows distinct responses of representative place fields in all the sessions. To quantify the rotation of all place fields fired in STD and MIS sessions, rotational correlation analysis was performed on all the active cells from all the rats. Figure 3C shows the circular histogram of STD vs. MIS and STD vs. STD comparisons. Place cells show stability in the STD sessions, exhibiting a significant mean vector length [MVL_(STD1 vs. STD2)_ = 0.89, MVL_(STD2 vs. STD3)_ = 0.89, Rayleigh’s test *p* < 0.01 in both comparisons]. During both mismatch sessions, place fields were oriented to either local or distal cues [MVL_(STD1 vs. MIS1)_ = 0.40, MVL_(STD2 vs. MIS2)_ = 0.64, Rayleigh’s test *p* < 0.01 in both comparisons].

**FIGURE 4:**
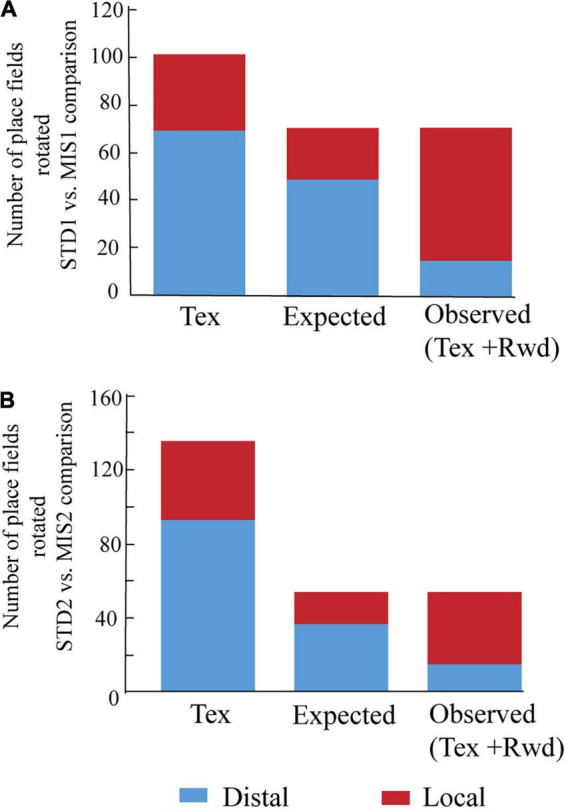
Comparison of place field orientation in the Tex and Tex + Rwd paradigm. **(A)** The stacked histogram of the number of place fields rotated with distal and local cues in STD1 vs. MIS1 comparison in the Tex paradigm with the expected and observed number of place field rotations in the Tex + Rwd paradigm. The number of place fields oriented to distal and local cues in the Tex paradigm is 69 and 32. In the Tex + Rwd paradigm, the observed numbers are 15 (distal) and 56 (local). **(B)** The stacked histogram of the number of place fields rotated with distal and local cues in STD2 vs. MIS2 comparison in the Tex paradigm with the expected and observed number of place field rotations in the Tex + Rwd paradigm. The number of place fields oriented to distal and local cues in the Tex paradigm is 93 and 43. In the Tex + Rwd paradigm, the observed numbers are 15 (distal) and 39 (local).

We then checked whether localizing reward alters the distribution of place field orientation (Figure 4). We found that, compared to Tex experiments, significantly higher number of place fields oriented toward the rotation of local cues in both MIS1 (Figure 4A, chi-square statistic with Yates correction = 31, *p* < 0.0001) and MIS2 (Figure 4B, chi-square statistic with Yates correction = 16.35, *p* < 0.0001) sessions in Tex + Rwd experiments. These results demonstrate that providing reward at a specific location biased the spatial representation of the hippocampus toward local cues.

Furthermore, these results also raise another interesting question–Can place cells orient their firing fields to reward features and locations? Two additional experiments were conducted to understand the specific role of different reward features like reward magnitude and reward flavor. In one set of experiments, rewards with different magnitudes were considered local cues. Different reward flavors act as local cues in the other set of experiments. In both these experiments, plain tracks were used, and the rewards were provided on visible reward locations (spokes) and were kept in conflict with distal cues in the cue-conflict sessions.

### Double Rotation Experiments With Different Reward Magnitude as Local Cues (RwdMag)

**FIGURE 5:**
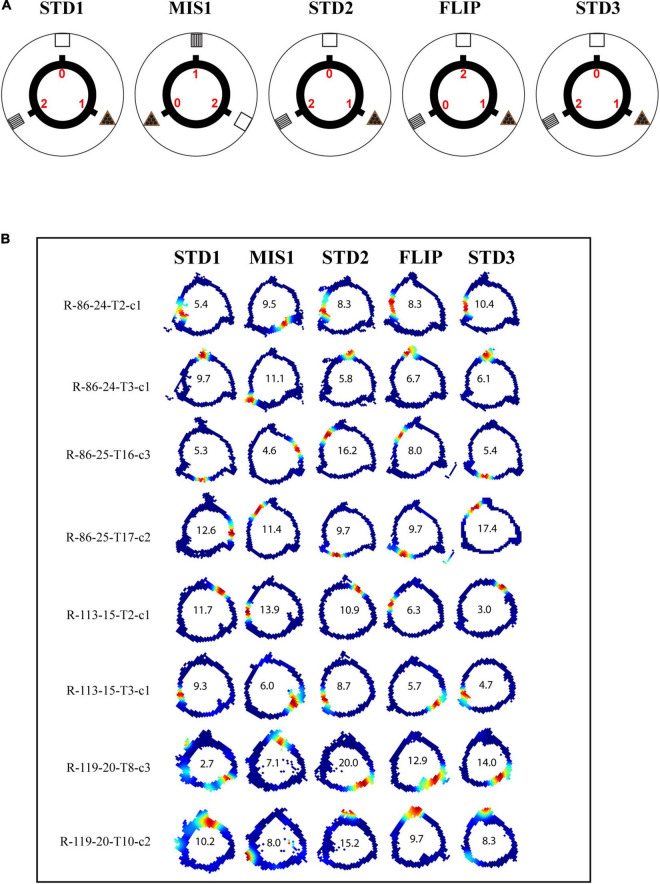
Schematic representation of double rotation experiments with different reward magnitude as local cues (RwdMag). **(A)** The schematic representation of the RwdMag double-rotation paradigm in which rewards of different magnitude were provided at specific spokes. Colored numbers (0, 1, 2) indicate the number of reward pellets offered at the corresponding spokes. **(B)** Representative place fields from different experimental days from five sessions. The rate map color coding is the same as described in Figure 2. The number inside the firing rate maps indicates the peak firing rate in Hz.

In this set of experiments, different reward magnitudes were kept in conflict with distal cues. The rats were trained to run CW on a plain black track with three spokes (black) separated 120° apart from each other. In this experiment, chocolate pellets (Bioserv, 45 mg) with different magnitudes were provided at specific spokes, and each spoke was associated with distinct distal cues. Here, rewards with different magnitudes act as local cues. Each day of the experimental session comprised three standard sessions (STD), separated by a mismatch session (MIS1) and a flip (FLIP) session. In the MIS1 session, the track was rotated CCW, and distal cues were rotated CW by 120°, leading to a change in the association between reward magnitude and distal cues. In the FLIP session, the distal cues were not rotated. However, the spoke, which provided a high reward, was provided with no reward, and the spoke, which provided no reward, was provided with a high reward. A total of 188 CA1 place cells were analyzed from four rats. The experimental paradigm is shown in Figure 5A.

Figure 5B shows place field orientation to different sessions from different rats. Out of 10 mismatch sessions, place fields rotated with the rotation of the track in 6 sessions, indicating that the reward magnitude as a local cue can control the orientation of place fields. The standard sessions were primarily stable (14/20 STD comparisons). During most FLIP sessions, place fields did not respond to a flip in magnitude as the position of the firing fields remained unchanged (7/10 sessions).

**FIGURE 6:**
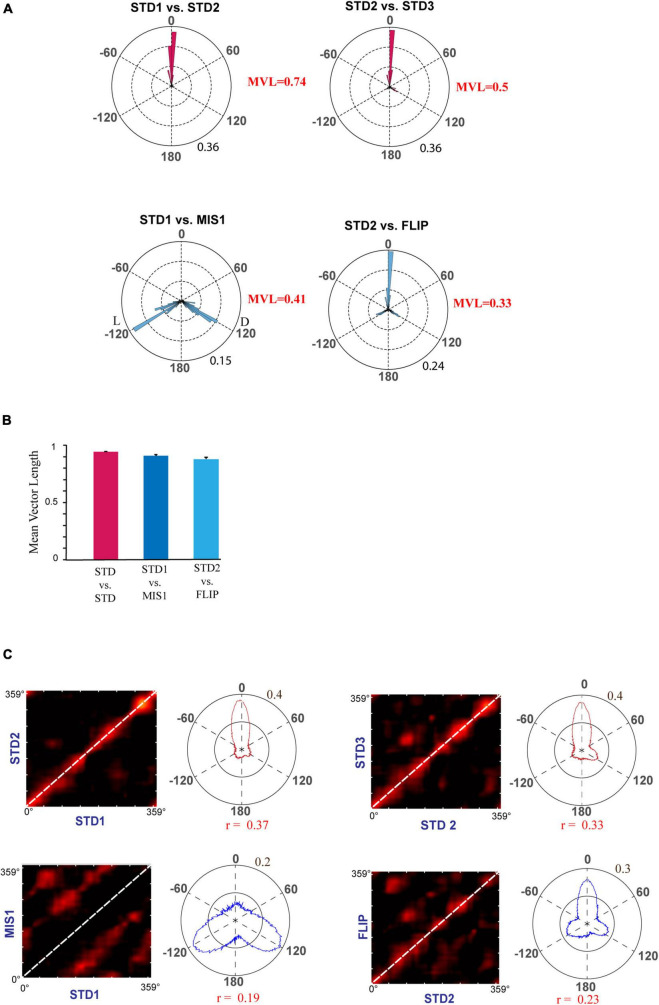
Representation of rotational correlation of place field population in the RwdMag experimental paradigm. **(A)** Circular histogram of rotational correlation of place fields in standard and mismatch/FLIP comparison. Mean vector length (MVL) of each comparison is shown near the corresponding plots. L and D indicate the rotation of local and distal cues. **(B)** Mean vector length of ensembles comparing STD and mismatch/FLIP comparisons. An error bar indicates the standard error of the mean. **(C)** Population correlation matrices of standard and mismatch/FLIP comparisons with the polar plot. The diagonal is marked with white-dotted lines. Transformation of the 2D correlation matrix to a 1D polar plot is presented alongside the correlation matrix. Maximum correlation value (r) of the polar plot is shown below the polar plot.

Figure 6A shows the circular histogram, depicting the rotation of place fields between STD and MIS/FLIP comparisons. Additionally, circular statistical analysis was performed on each ensemble across comparisons to study the rotation of place fields of all the simultaneously recorded neurons by calculating their mean vector length. Average MVL of co-recorded neurons in STD sessions suggests that the rotation of place fields is highly coherent [MVL_(STD1 vs. STD2)_ = 0.93, *p* < 0.001; MVL_(STD2 vs. STD3)_ = 0.86, *p* < 0.001]. MVL values of MIS1 [MVL_(STD1 vs. MIS1)_ = 0.91, *p* < 0.001] and FLIP [MVL_(STD2 vs. FLIP)_ = 0.88, *p* < 0.001] sessions also indicate that place fields from all the co-recorded neurons exhibited a coherent rotation (Figure 6B). Population correlation matrices also show stability in STD sessions. Correlation matrices of MIS comparison showed bands at the angle of rotation of both distal and local cues, indicating the dynamic and coherent structure in the hippocampal representation. Still, the hippocampal representation was more stable during the FLIP comparison (Figure 6C). These results suggest that the hippocampus maintained a coherent representation, but the orientation of place field assemblies is dynamic as, on some days, the STD sessions were not stable. However, the place cells did not alter their field location during most recording days when the reward values were interchanged without rotating the distal cues.

### Double Rotation Experiments With Different Reward Flavors as Local Cues (RwdFlav)

**FIGURE 7:**
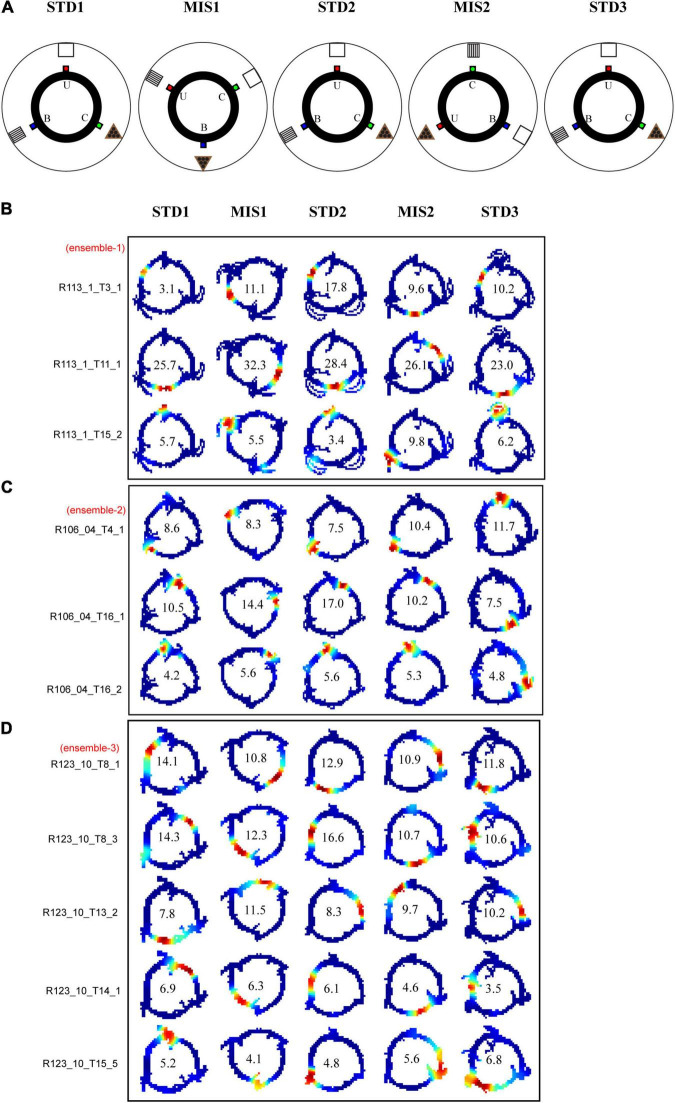
Schematic representation of double rotation experiments with different reward flavors as local cues (RwdFlav). **(A)** The schematic representation of the double rotation paradigm using rewards of different flavors as local cues. The blue dot indicates the spoke where banana flavor pellets (B) were provided, the red dot indicates the spoke where sucrose pellets (U) were provided, and the green dot indicates the spoke where chocolate-flavored pellets (C) were provided. **(B–D)** Representative place fields from different experimental days from five sessions. On the left side, cluster-ID is provided. The number inside the firing rate maps indicates the peak firing rate in Hz.

In this set of experiments, three distinct reward flavors act as local cues. The rats were trained to run CW on a plain black track with three spokes (black) separated 120° apart (the same track as RwdMag experiments). Three different flavors of pellets, namely, sucrose, chocolate, and banana-flavored pellets (Bioserv Dustless Precision pellets, 45 mg), served as rewards and were manually delivered at each specific spoke. In this experiment, reward flavors were considered local cues as the animals directly experienced these cues. Each day of the experimental session comprised three standard sessions (STD) separated by two mismatch sessions (MIS). The experimental paradigm is shown in Figure 7A. We analyzed 591 place cells recorded from five rats.

**FIGURE 8:**
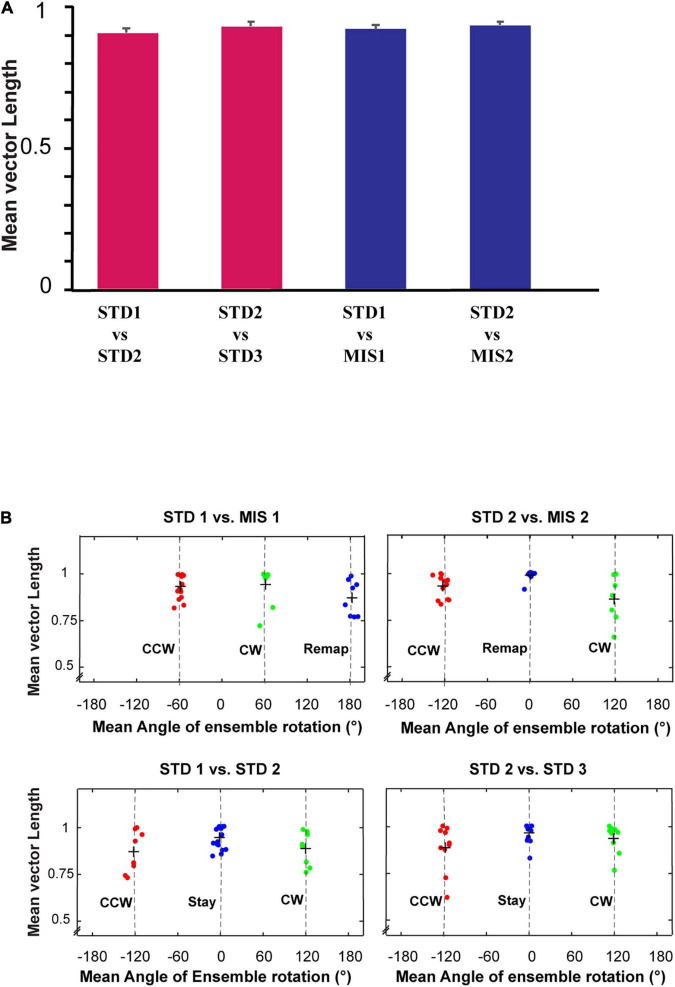
Mean vector length distribution and dynamics of place cell orientation. **(A)** Mean vector length of co-recorded cells (ensemble) in different standard and mismatch comparisons. An error bar indicates the standard error of mean. **(B)** A scatter plot of the mean direction and mean vector length of all the ensembles across standard and mismatch sessions. Red, green, and blue dots show the clustering of the data points based on k-means elbow method. “+” indicates the centroid of each cluster. In standard vs. mismatch comparisons, the red cluster signifies the ensembles that followed the rotation of CCW reward flavors (CCW), the green cluster signifies the ensembles that followed the rotation of CW distal cues (CW), and the blue cluster signifies the ensembles that rotated away (Remap), not following either reward flavors or distal cues. In standard vs. standard comparisons, the red cluster signifies the ensembles that followed the net CCW rotation (CCW), the green cluster signifies the ensembles that followed the net CW rotation (CW), and the blue cluster signifies the ensembles that showed a stable representation between standard sessions (stable). Gray-dotted line denotes the angle of separation of spokes/cues (120°).

Figures 7B–D show the distinct orientation of place field ensembles. The place field rotation indicates that, even though we did not observe the place field stability in some of the STD sessions, all the place cells coherently shifted their fields (see Supplementary Figure 1 for all the co-recorded neurons in Ensemble 1 from Figure 7B). This result shows the dynamic orientation of place cell ensembles during the mismatch sessions. Figure 8A shows the MVL across STD vs. MIS and STD vs. STD comparisons for all the ensembles. The average MVLs across ensembles in the pooled STD1 vs. MIS1, STD2 vs. MIS2, STD1 vs. STD2, and STD2 vs. STD3 comparisons are 0.921 ± 0.015, 0.933 ± 0.014, 0.907 ± 0.015, and 0.930 ± 0.016, respectively (mean ± S.E.M.). These results suggest that the peak angles of correlation of individual neurons in each ensemble across all the comparisons were clustered in one particular direction (in a particular session). Across all the comparisons, the average MVL is high and statistically significant (>0.8, Lee et al., 2004; Rayleigh test: *p* < 0.01), indicating a coherent structure in the orientation.

Individual ensembles across comparisons that exhibited a coherent structure with a high mean vector length also showed distinct orientation across sessions. We investigated if any underlying pattern exists in these ensembles. We used the k-means algorithm to optimize the number of clustered ensembles obtained based on each ensemble’s mean vector length and mean direction across sessions. Figure 8B shows the scatter plot of the ensemble mean vector length and mean direction with three clusters across STD vs. STD and STD vs. MIS comparisons. Each cluster group has ensembles orientated toward a specific cue rotation where CCW and CW groups were aligned with CCW and CW rotation of reward flavor and distal cues, respectively. The Remap group is located equidistant from CCW and CW groups (∼120°), indicating the spacing of the orientation of the ensembles based on the angle of separation between the spokes. Many ensembles showed a more stable response (stay) in STD vs. STD comparisons. In STD vs. MIS conditions, more ensembles oriented toward the rotation of reward flavors than the distal cues [48% CCW (∼ -60°), 27% CW (∼60°), and 24% has remapped (∼180°) in STD1 vs. MIS1 comparison, 42% CCW (∼ -120°), 24% CW (∼120°), and 34% remapped (∼0°) in STD2 vs. MIS2 comparison].

This result shows the place fields did not follow the rotation of reward flavors or distal cues under some sessions but were rapidly reorienting with spokes. However, the orientation is not just random, as, in 21% of the experimental days, the place fields oriented to one set of cues only (Chi-square statistic, 9.12, *p* < 0.002). This result shows that the orientation of ensembles in a cue mismatch environment is essentially dynamic and slightly biased toward the reward cues. On other days (across rats), the ensembles showed a dynamic shift in orientation (in at least one session) from one cue to another or shifted based on the position of spokes. We observed a dynamic shift in the orientation during some standard sessions, leading to a reorientation of place fields. However, the place fields coherently rotated away from the initial location even during this rapid reorientation. The cluster groups obtained organized the ensembles into three groups in all STD vs. STD and STD vs. MIS comparisons, and rotational analysis was performed in the entire neural population in each group. All the active neurons were pooled together from each ensemble in a particular cluster group from their respective STD vs. STD and STD vs. MIS comparisons. Circular statistics were performed on all ensemble data from each group, and the distribution also showed a coherent orientation (Supplementary Figure 2).

**FIGURE 9:**
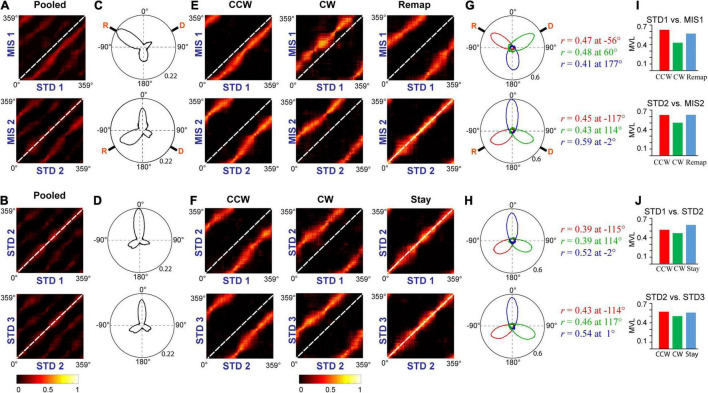
Population response in the hippocampus. **(A,B)** Correlation matrices from population firing rate vectors of all the cells pooled from each ensemble at each position on the track between STD vs. MIS **(A)** and STD vs. STD sessions **(B)**. **(C,D)** The hippocampal population activity from panels **(A,B)** is represented as polar plots in panels **(C,D)**, respectively. **(E,F)** Grouped correlation matrices from population firing rate vectors of all the cells present in each cluster of ensembles at each location on the track between STD vs. MIS (CCW, CW, and Remap) and STD vs. STD sessions (CCW, CW, and Stay). **(G,H)** The hippocampal population activity of different groups between STD vs. MIS sessions [CCW (red), CW (green), and Remap (blue)] and STD vs. STD sessions [CCW (red), CW (green), and Stay (blue)] are represented as polar plots (constructed from correlation matrices). The values next to the polar plot indicate the angle of peak correlation (r) of the hippocampal population at corresponding groups. **(I,J)** Length of the mean vector in each group in STD vs. MIS and STD vs. STD sessions for each of the corresponding polar plots is shown in panels **(G,H)**.

Furthermore, we conducted population correlation analysis to measure the extent of CA1 coherency across each position on the track (Figures 9A,B). Polar plots of the population correlation matrices showed three peaks separated ∼120° apart (Figures 9C,D). When pooled using the k-means method, CA1 showed a band of high correlation (Figures 9E,F). These suggest that, even though the population response of the hippocampus was unstable across some STD sessions and exhibits dynamic orientation in MIS sessions, all the cells rotated as an ensemble under all the conditions, thus demonstrating a location-specific coherent population activity. The polar plots also show the presence of a single peak in all the groups across comparisons (Figures 9G,H). Figures 9I,J show the mean vector length of the polar plots at each group and STD vs. MIS and STD vs. STD comparisons, respectively. Also, there is no significant difference in the mean vector length across different comparisons (Kruskal–Wallis test, *p* = 0.826), indicating similar responses in both STD vs. STD and STD vs. MIS comparisons. These results show that the hippocampus maintains a location-specific coherency despite the dynamic change in the orientations. We have also checked whether reward alters the distribution of place fields across the tracks. We found that the place field distribution was not uniform as significantly more place fields were present at the reward locations (Supplementary Figure 3), which is in line with other studies (Hollup et al., 2001).

### Adding Textures Increases the Stability of the Hippocampal Representation

**FIGURE 10:**
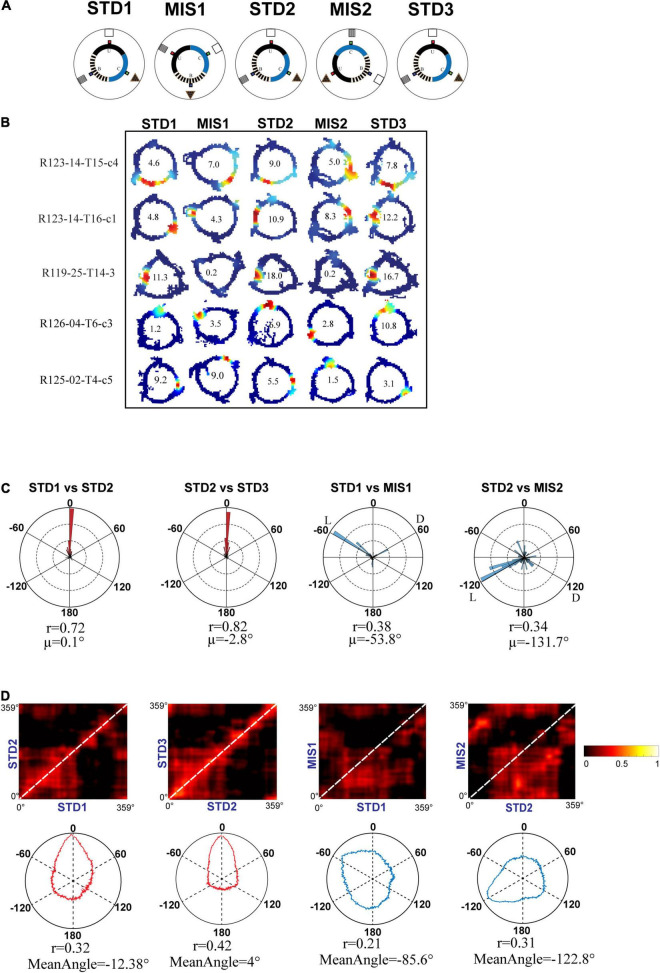
Schematic representation of double rotation experiments with different reward flavors and surface textures as local cues (RwdFlav + Tex). **(A)** Schematic representation of the reward flavor + texture–a distal cue double-rotation paradigm. The blue dot indicates the spoke where banana flavored-pellets (B) were provided, the red dot indicates the spoke where sucrose pellets (U) were provided, and the green dot indicates the spoke where chocolate-flavored pellets (C) were provided. **(B)** Representative place fields from different experimental days from five sessions. **(C)** Circular histogram of place field rotation in standard and mismatch comparisons. L and D indicate the rotation of local and distal cues. **(D)** Population correlation matrices and the corresponding polar plot of standard and mismatch comparisons.

Even though the hippocampus showed coherent representation in RwdFlav experiments, in some STD sessions, place field reorientation was observed. We conducted an additional set of experiments in which textured tracks (RwdFav + Tex) were introduced in the same experimental paradigm (Figure 10A). We hypothesized that adding textures to the tracks would make the hippocampal system more stable in the STD sessions than RwdFlav experiments, where only reward flavors were provided. A total of 108 CA1 place cells from 4 rats were used for the analysis. Figure 10B shows representative examples of neurons from all five sessions. Circular histogram of rotation of place fields in different comparisons shows that STDs were more stable (Figure 10C). Also, this suggests that more neurons were oriented to the local cues than the distal cues, same as that observed in Text + Rwd experiments. The population correlation matrices also indicate that local texture cues provided stability to the place fields in STD sessions (Figure 10D).

### Distinct Network Dynamics in the Hippocampus

**FIGURE 11:**
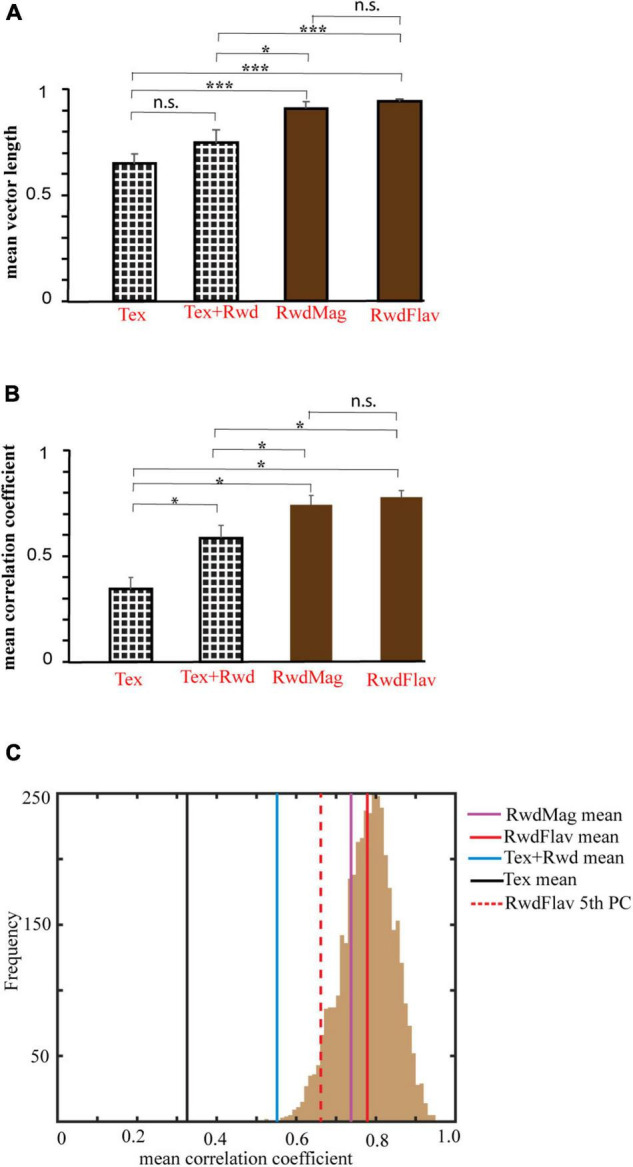
Comparison of mean vector length and correlation coefficient across different experiments. **(A)** Mean vector length of mismatch sessions in different experimental paradigms. The error bar indicates the standard error of the mean. n.s indicates non-significant. * indicates *p* < 0.02, *** indicates *p* < 0.001 (Kruskal–Wallis test, Bonferroni corrected). **(B)** Mean correlation coefficient obtained from a spatial coupling between subsequent sessions in different experimental paradigms. * indicates *p* < 0.05, n.s. indicates non-significant. (Kruskal–Wallis test, Bonferroni corrected). **(C)** Histogram of coefficient of correlation calculated by the bootstrap sampling of any random 200 cell pairs between adjacent sessions (1,000 repetitions) in the RwdFlav experiment. The red-dotted line and the solid red line indicate the 5th percentile and the mean of the correlation coefficient obtained (RwdFlav experiments). Mean correlation coefficients of other experiments obtained by bootstrap sampling are also shown. The mean correlation of Tex and Tex + Rwd experiments is lower than the 5th percentile of RwdFlav experiments (*p* < 0.05).

We next investigated the nature of network dynamics underlying these representations by studying the coherency of the hippocampal systems in different double rotation experiments. First, we averaged all the ensembles’ mean vector length in all rotations across the experiments. Figure 11A shows that co-recorded hippocampal neurons in RwdMag and RwdFlav experiments exhibited significantly higher mean vector length (*p* < 0.02, Kruskal–Wallis test, after Bonferroni correction), indicating more coherency in the presence of reward, possibly suggesting an attractor-like representation in the hippocampus. A spatial or head direction system exhibits an attractor-like network when the neurons maintain their spatial offset across sessions. We then calculated the mean direction of co-recorded neurons across subsequent sessions to study the spatial offset between different experiments. Figure 11B shows that the mean correlation coefficient is significantly higher (*p* < 0.05, Kruskal–Wallis test, after Bonferroni correction) when reward features alone were presented as local cues. This result indicates that co-recorded neurons maintained spatial offset under these conditions (see Supplementary Figure 4 for the density scatter plot). A bootstrapping procedure was performed to verify whether the significance observed is due to the difference in cell pairs across the experiments. Two hundred random samples were taken with repetition from each comparison and computed their mean correlation coefficient. This procedure was repeated 1,000 times across experiments. The mean correlation coefficient for each experiment was computed and compared across the 5th percentile of RwdFlav experiments. The experiments using the textured track have shown that reward features alone have a significantly high mean correlation coefficient (bootstrap *p* < 0.05), indicating a coherent structure (Figure 11C). These results demonstrate that the hippocampus uses a single coherent attractor-like spatial map in reward-distal cue mismatch environments. In contrast, the coherency was reduced in a dynamically changing environment with local textures, and the hippocampus established context-specific spatial maps under these conditions.

## Discussion

In this study, we used a series of experiments to study whether different reward features modulate the hierarchical spatial representation of the hippocampus. First, we conducted classical double rotation experiments (Tex) and replicated the finding that co-recorded place cells exhibited heterogeneous responses. Then, we ran the experiments with localized reward (Tex + Rwd). We found that, compared to Tex experiments, a significantly higher number of place fields were controlled by the local cues, suggesting the prominent role of reward in modulating the spatial orientation of the hippocampal place cells. Additional experiments (RwdMag and RwdFlav) were conducted to study how specific reward features modulate the hippocampal population. In these experiments, reward magnitudes and reward flavors act as local cues. In both conditions, the hippocampus maintained a coherent and dynamic response. We observed coherent rotation of place fields, following the rotation of either reward features or distal cues in many sessions. On many days, the place fields were only controlled by reward features. On some occasions, the place fields were oriented away based on the reward location. Still, they exhibited coherency, indicated by the maintenance of spatial offset by co-recorded neurons. The spatial offset is not maintained when surface textures act as local cues (Tex). However, introducing textured tracks increases the stable firing of place cells in standard sessions (RwdFlav + Tex), implying the role of surface textures in recognition memory. Our novel findings indicate distinct functioning of CA1 as an attractor-like network under conditions when only localized reward features were presented as local cues in visible reward locations.

Previous studies have shown that CA1 exhibited diverse responses in cue conflict experiments. The place fields from co-recorded CA1 neurons rotated with distal cues, local cues, appear, disappear, remap, or even show split representations (Knierim, 2002; Lee et al., 2004). Anatomically, CA1 integrates spatial knowledge *via* EC (entorhinal cortex) and CA3 pathways, providing information about the organism’s current state and mnemonic representation (Witter, 1993). The heterogeneous response in CA1 during cue-conflict conditions is due to different inputs arising from EC and CA3. This change in computation generally enables CA1 to function as a comparator or a mismatch detector that compares the spatial representation of EC (current state) and mnemonic representation cached in the CA3 (Duncan et al., 2012; Knierim and Neunuebel, 2016).

However, experiments with localized reward biased more neurons to orient their place fields in the direction of rotation of local cues. A plausible explanation for the biased response is that providing reward at a specific location skewed the animal’s attention to the track or track-based cues. Studies have also found that the hippocampus comprises dedicated cells that encode rewards and goals (Sosa and Giocomo, 2021). In a cue-conflict condition, the activity of goal and reward cells might pull the activity of nearby neurons, resulting in more neurons firing toward the direction of the rotation of local cues.

Recent studies have suggested that the hippocampus integrates reward information into the spatial processing framework (Aoki et al., 2019; Xiao et al., 2020). Hippocampal place fields are shown to accumulate at reward or goal locations (Hollup et al., 2001; Hok et al., 2007). Distinct reward-based circuits in the hippocampus recognize a change in context and reward information (Luo et al., 2011; Kaufman et al., 2020). Also, studies have demonstrated that learning of specific goals distorted entorhinal grid maps toward the goal and altered MEC cells’ firing rate (Boccara et al., 2019; Butler et al., 2019). Based on double rotation experiment results (Knierim, 2002), when the association between reward features and distal cues was altered, it was expected that CA1 would exhibit heterogeneous activity. Strikingly, we did not observe such response in the experimental paradigms in which reward features (flavors and magnitudes) and distal cues were rotated. Instead, CA1 ensembles exhibited coherency, even during reorientation, suggesting the interplay of attractor network dynamics. The coherent response might be because of the subthreshold change in EC and CA3 input to CA1 in dynamically changing environments. Under this condition, CA1 might not receive conflicting information and thus could not function as a comparator to differentiate contexts (Barrientos and Tiznado, 2016). Therefore, CA1 might relay the output from the recurrent collateral auto-associative CA3 network, featuring coherent spatial representation (Rolls, 2007; Knierim and Zhang, 2012; Neunuebel and Knierim, 2014).

One primary reason behind the coherent response might be the absence of continuous local cues. Here, reward features were provided only at the spokes. Thus, the discretization of the local cue experience may not differentiate the EC and CA3 representations in cue-conflict sessions, leading to the coherent representation of CA1. Also, it is essential to note that the cues used in classical double rotation studies (Knierim, 2002) are neutral. On the other hand, reward features can skew the animal’s attention more to the reward or track-based cues like spokes. Our results show this inclination, as the hippocampal place fields are oriented to reward features under many experimental days. Also, our results in RwdFlav and RwdMag experiments show that the clustering of orientation angle is ∼120° apart, showing that place field orientations correspond to the reward locations. The non-neutral feature of reward and the presence of spokes as a cue might be one of the reasons that can explain the reorientation in the standard sessions. These results also demonstrate that track geometry may guide spatial orientation (Keinath et al., 2017).

Recent studies have shown that place field maps show random and coherent rotation in an exploration task without orienting to specific arena cues (Kinsky et al., 2018; Muzzio, 2018). Our work also shows dynamic coherent rotation, but the task is more goal directed, having specific rewards features, requiring the animals to run in a particular direction for food rewards. However, when surface textures were introduced, we could observe the stability in the representation of hippocampal neurons, suggesting the use of multisensory information for place field stability (Save et al., 2000). The local surface textures enabled the CA1 to differentiate it as different contexts in dynamically changing environments. Recent studies have also shown that the hippocampal place cells encode the boundaries of local surface textures (Wang et al., 2020), and spatial coding resolution was increased in the presence of local visual cues (Bourboulou et al., 2019).

In conclusion, we showed that, during goal-directed behavior to learned reward position, attractor-like dynamics control the hippocampal CA1 activity. The present study shows a distinct computation in the hippocampus that exhibits attractor dynamics in the presence of reward features. But the reward features alone could not create a context-specific spatial map as it may not substantially impact pattern separation due to subthreshold difference in input from CA3 and EC. The heterogeneous response is observed when surface textures were used as local cues, indicating the role of visual/tactile features in categorizing and recognizing environmental contexts. Our study also suggests that the animal’s attention to the external cues and the nature of stimuli in the environment dictates the dynamics of spatial representation in the hippocampus.

## Data Availability Statement

The raw data supporting the conclusions of this article will be made available by the authors, without undue reservation.

## Ethics Statement

The animal study was reviewed and approved by Institutional Animal Ethics Committee (IAEC) of National Brain Research Center at Manesar, Haryana, constituted by the Committee for the Purpose of Control and Supervision of Experiments on Animals (CPCSEA), Government of India.

## Author Contributions

IN conceived and designed the study and performed the data analysis. IN and GB performed the experiments and histology. IN and DR interpreted the data and wrote the manuscript. All authors revised and edited the manuscript.

## Conflict of Interest

The authors declare that the research was conducted in the absence of any commercial or financial relationships that could be construed as a potential conflict of interest.

## Publisher’s Note

All claims expressed in this article are solely those of the authors and do not necessarily represent those of their affiliated organizations, or those of the publisher, the editors and the reviewers. Any product that may be evaluated in this article, or claim that may be made by its manufacturer, is not guaranteed or endorsed by the publisher.

## Supplementary Material

Supplementary Figure 1 Rate maps of co-recorded neurons from Ensemble 1 (RwdFlav). Rate maps of all the neurons present in Ensemble 1 across STD and MIS sessions from Rat-113. Ensemble 1 has 27 co-recorded neurons. The number inside the firing rate maps indicates the peak firing rate in Hz.

Supplementary Figure 2 Dynamic and coherent representation in the CA1 in the RwdFlav paradigm. **(A,B)** The amount of rotation of firing fields of all the active place cells in all the ensembles in the CCW group (the red circle), the CW group (the green circle), and the AR group (the blue circle) between STD vs. MIS sessions, represented around the circle. **(C,D)** The amount of rotation of firing fields of all the active place cells in all the ensembles in the CCW group (the red circle), the CW group (the green circle), and the Stay group (the blue circle) between STD vs. STD sessions, represented around the circle. The direction of arrow represents the mean angle of rotation of population of neurons in each group, and length of the arrow signifies the compactness of the distribution around the mean angle. The brown line indicates the rotation angle of reward flavors (R) and distal cues (D) in MIS sessions (circle; open = 1 cell, filled = 5 cells). Values of mean angle of the distribution, 95% confidence interval, length of the mean vector (r) and its significance level (p) are shown near to each plot.

Supplementary Figure 3 **(A)** Histogram of the peak firing position of the place cells in all the sessions obtained by linearizing the track and binning the track into 72 bins (5° per bin). The dotted line indicates the position of the spokes. U, B, and C indicate the spokes that provided the unflavored, banana-flavored, and chocolate-flavored pellets. **(B)** Chi-Square values for each session when compared with expected values.

Supplementary Figure 4 Density scatter plots of the mean direction of place cell pairs after spatial cross-correlation of co-recorded neurons between adjacent sessions in Tex (*N* = 344), Tex + Rwd (*N* = 643), RwdMag (*N* = 1,305), and RwdFlav (*N* = 5,288) experimental paradigms. In the color map, blue indicates 0, and red indicates the maximum number of cell pairs in a given experiment.
