# Supplementary figures and images for: Hippocampus Maintains a Coherent Map Under Reward Feature–Landmark Cue Conflict
Source: Front Neural Circuits. 2022 Apr 26;16:878046. doi: 10.3389/fncir.2022.878046 (PMC9086833; doi:10.3389/fncir.2022.878046)

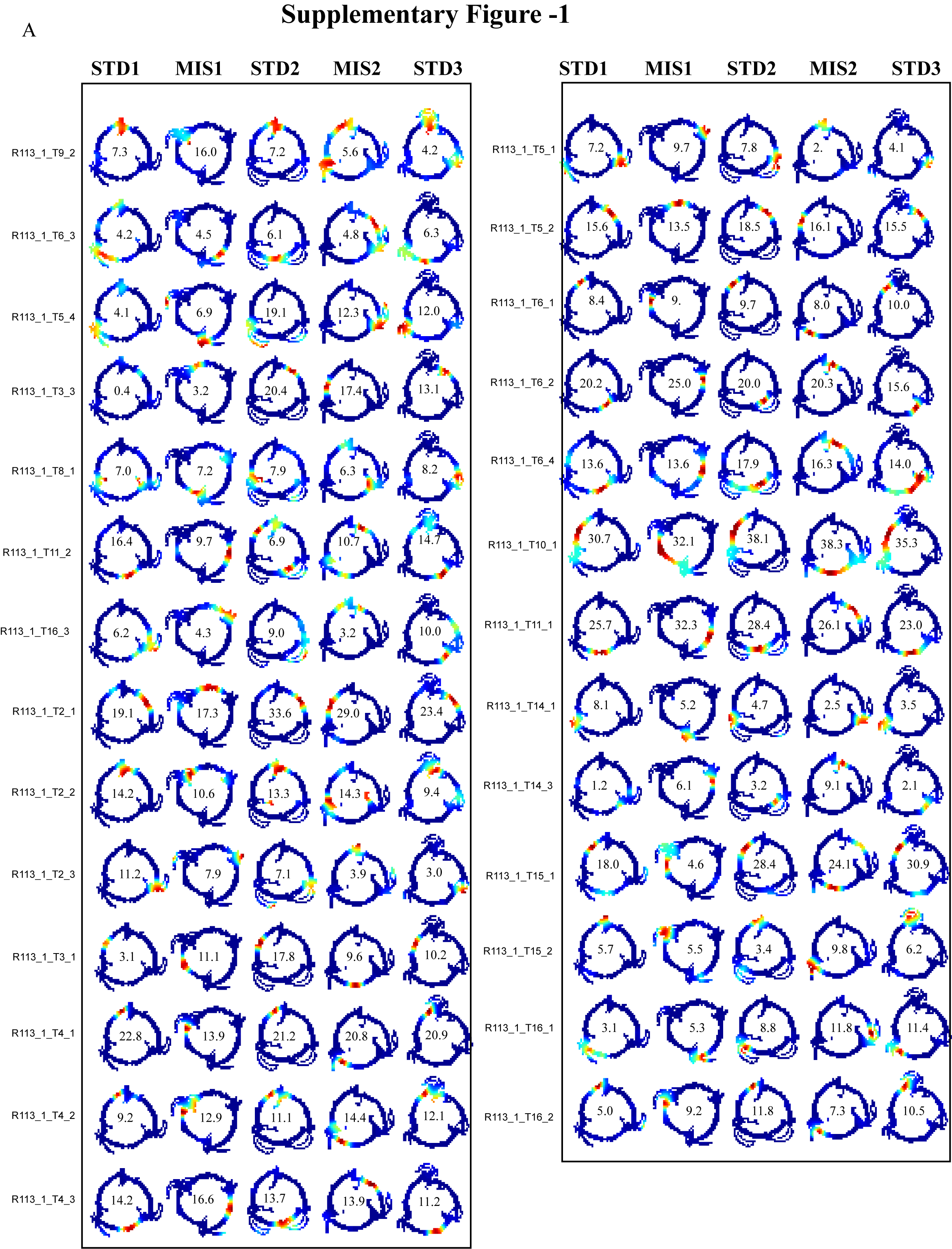

Supplement: Supplementary Figure 1 — Rate maps of co-recorded neurons from Ensemble 1 (RwdFlav). Rate maps of all the neurons present in Ensemble 1 across STD and MIS sessions from Rat-113. Ensemble 1 has 27 co-recorded neurons. The number inside the firing rate maps indicates the peak firing rate in Hz. [file Image_1.TIF]

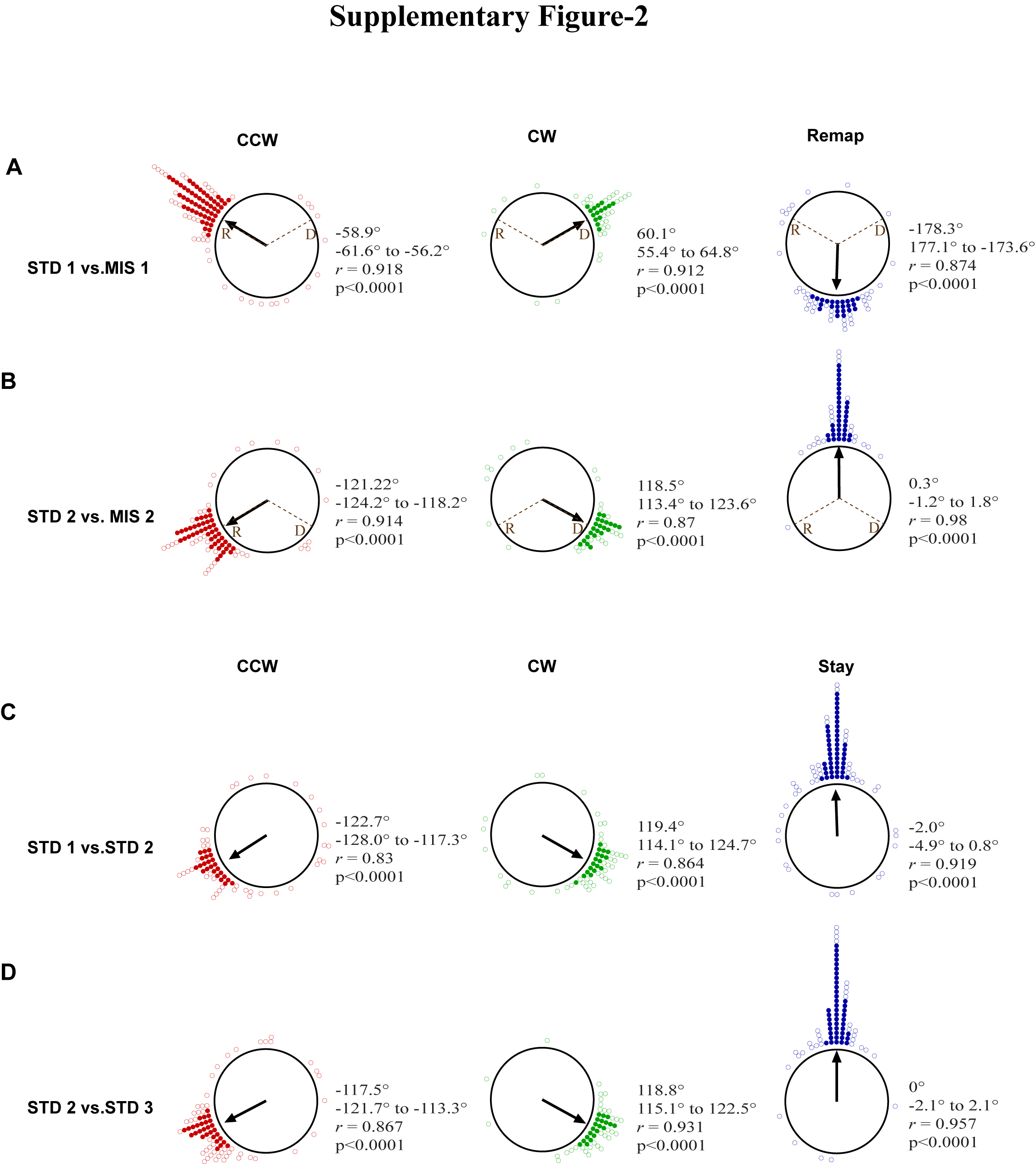

Supplement: Supplementary Figure 2 — Dynamic and coherent representation in the CA1 in the RwdFlav paradigm. (A,B) The amount of rotation of firing fields of all the active place cells in all the ensembles in the CCW group (the red circle), the CW group (the green circle), and the AR group (the blue circle) between STD vs. MIS sessions, represented around the circle. (C,D) The amount of rotation of firing fields of all the active place cells in all the ensembles in the CCW group (the red circle), the CW group (the green circle), and the Stay group (the blue circle) between STD vs. STD sessions, represented around the circle. The direction of arrow represents the mean angle of rotation of population of neurons in each group, and length of the arrow signifies the compactness of the distribution around the mean angle. The brown line indicates the rotation angle of reward flavors (R) and distal cues (D) in MIS sessions (circle; open = 1 cell, filled = 5 cells). Values of mean angle of the distribution, 95% confidence interval, length of the mean vector (r) and its significance level (p) are shown near to each plot. [file Image_2.TIF]

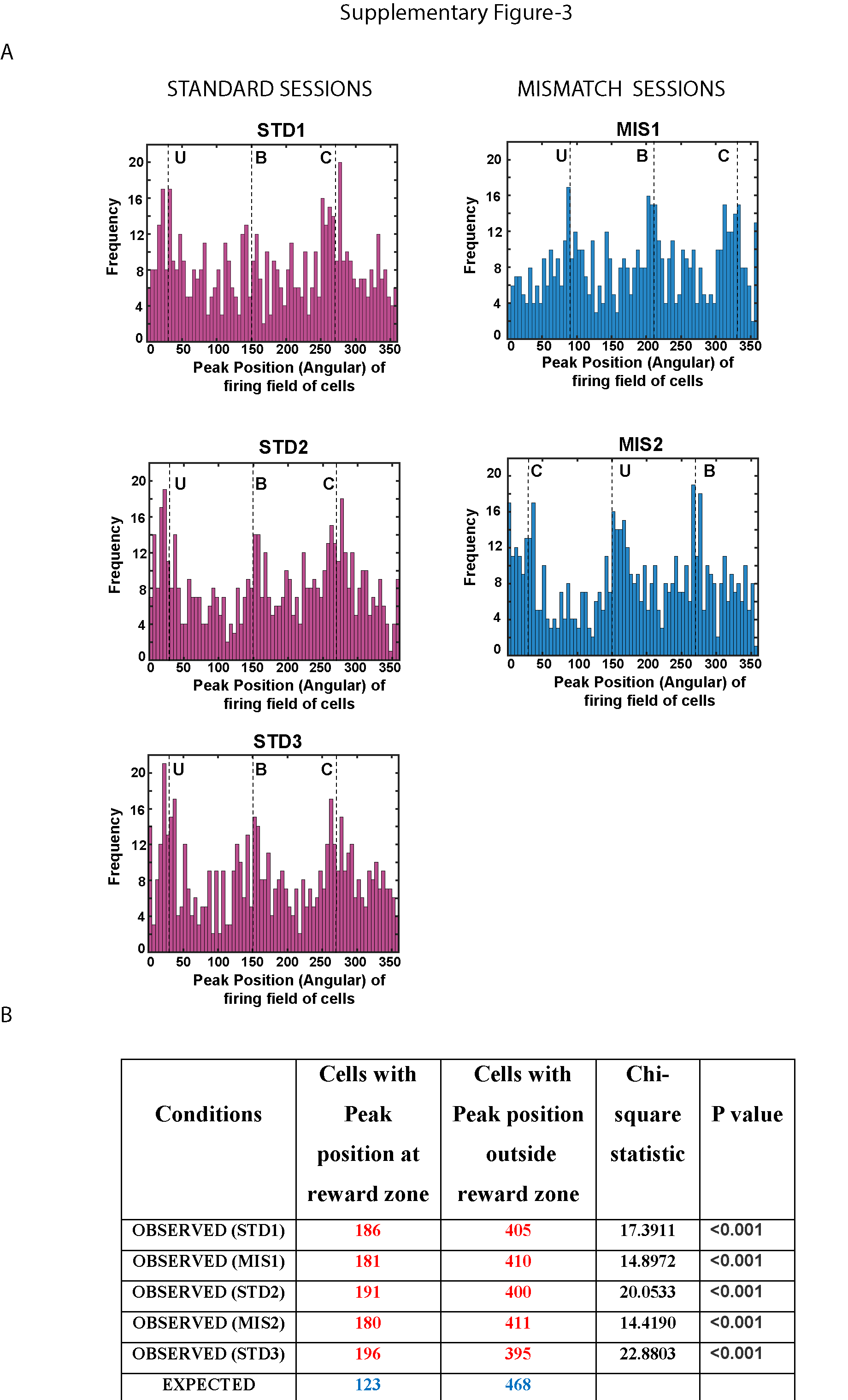

Supplement: Supplementary Figure 3 — (A) Histogram of the peak firing position of the place cells in all the sessions obtained by linearizing the track and binning the track into 72 bins (5° per bin). The dotted line indicates the position of the spokes. U, B, and C indicate the spokes that provided the unflavored, banana-flavored, and chocolate-flavored pellets. (B) Chi-Square values for each session when compared with expected values. [file Image_3.TIF]

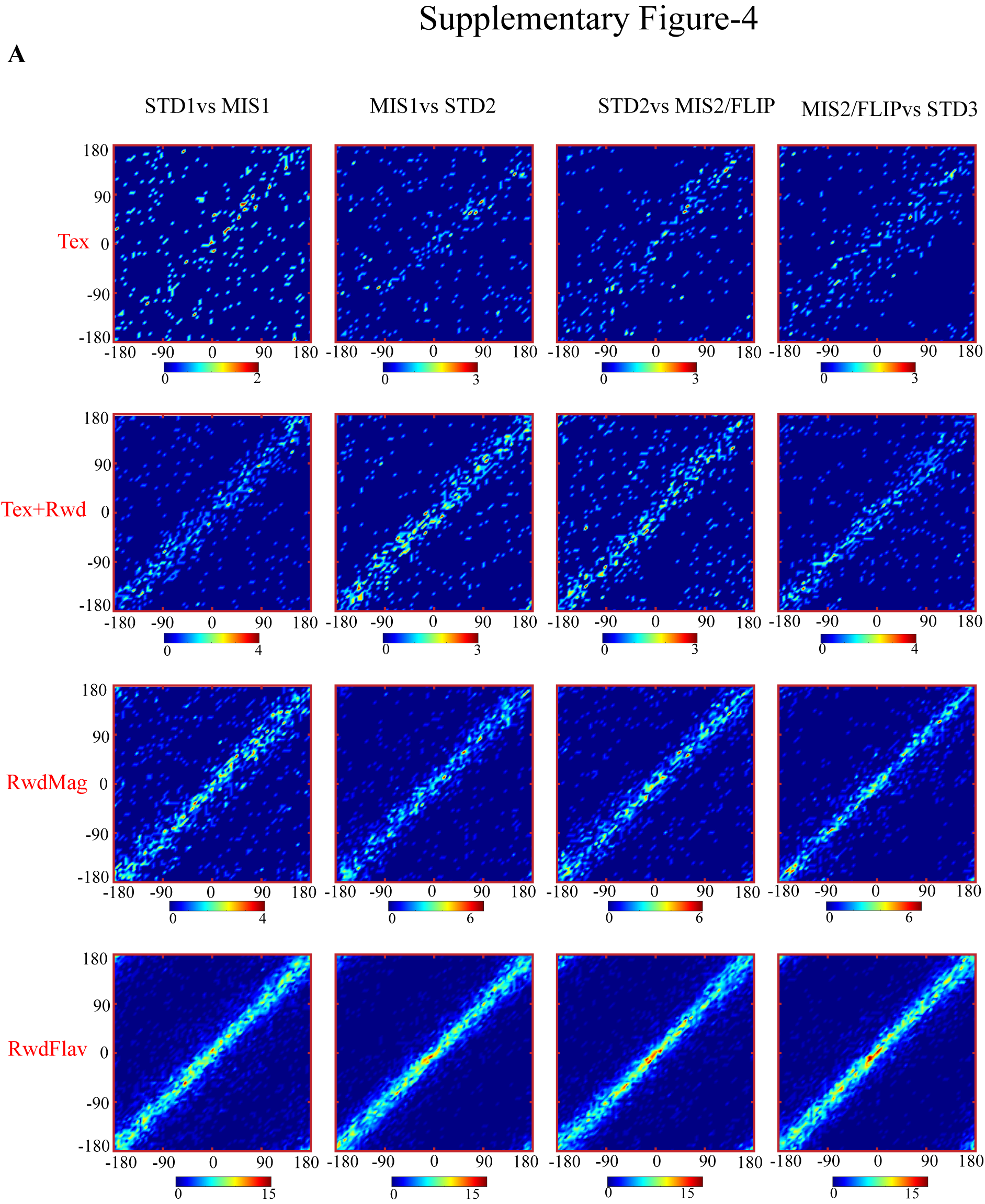

Supplement: Supplementary Figure 4 — Density scatter plots of the mean direction of place cell pairs after spatial cross-correlation of co-recorded neurons between adjacent sessions in Tex (N = 344), Tex + Rwd (N = 643), RwdMag (N = 1,305), and RwdFlav (N = 5,288) experimental paradigms. In the color map, blue indicates 0, and red indicates the maximum number of cell pairs in a given experiment. [file Image_4.TIF]
